# The Gene *SiPrx* from *Saussurea involucrata* Enhances the Stress Resistance of *Silphium perfoliatum* L.

**DOI:** 10.3390/plants14071030

**Published:** 2025-03-26

**Authors:** Tao Liu, Baotang Wu, Yao Zhang, Zhongqing Li, Yanhua Xue, Xiaoqin Ding, Zhihui Yang, Jianbo Zhu, Yajie Han

**Affiliations:** 1 College of Life Science, Shihezi University, Shihezi 832003, China; 13405579677@163.com (T.L.); 17623691363@163.com (B.W.); zhangyaolzq@163.com (Y.Z.); mzzxqlzq@163.com (Z.L.); 13683771408@163.com (Y.X.); dingxiaoqin0122@163.com (X.D.); shzyangzhihui@163.com (Z.Y.); 2 School of Chemistry and Chemical Engineering, Shihezi University, Shihezi 832003, China

**Keywords:** *Saussurea involucrata*, *SiPrx*, transgenic, *Silphium perfoliatum* L., salt stress, cold stress

## Abstract

Peroxiredoxin (Prx) plays a role in maintaining the balance of intracellular reactive oxygen species. The peroxidase *SiPrx* gene from the Tianshan Snow Lotus (*Saussurea involucrata*) has been proved to significantly enhance the stress resistance of plants. In this study, the *SiPrx* gene was expressed heterogeneously in high-quality herbage *Silphium perfoliatum* L. (SP). After treatment with NaCl, the transgenic SP only exhibited partial leaf wilting, whereas the wild-type (WT) plants were on the brink of death. Simultaneously, physiological and biochemical assays indicated that under high-salt conditions, the content of malondialdehyde in the transgenic plants was significantly lower than that in the WT plants, while the activity of antioxidant enzymes was significantly higher than that in the WT plants. The expression of the *SiPrx* gene has been shown to significantly enhance the salt stress resistance of transgenic SP. Furthermore, after treatment at −10 °C for 48 h, the leaves of transgenic plants were able to maintain a certain morphological structure, whereas the WT plants were completely wilted. Physiological and biochemical index measurements indicated that all indicators in the transgenic plants were significantly better than those in the WT plants. Based on these findings, this study plans to overexpress the *SiPrx* gene extracted from *Saussurea involucrata* in Comfrey using the Agrobacterium-mediated method and then study its effects on the stress resistance of transgenic SP. The research results indicate that the *SiPrx* gene shows significant application potential in enhancing the cold resistance and salt tolerance of SP. This study provides a certain research basis and scientific evidence for the mining of stress resistance genes in *Saussurea involucrata* and the cultivation of new varieties of SP.

## 1. Introduction

Reactive oxygen species (ROS) are normal products of cellular metabolism, and their main forms existing within cells are primarily hydrogen peroxide (H_2_O_2_), hydroxyl radicals (HO·), superoxide anions (O^2−^), etc. [1]. When plants are under stress, the accumulation of ROS will lead to an imbalance of the cell membrane system. A high level of ROS can directly or indirectly cause DNA damage [2] and lipid and protein peroxidation [3]. In order to maintain the balance of ROS and avoid the damage caused by oxidative stress, there is a complex antioxidant system in organisms, and peroxidases (PRXs) are one of them. PRXs are a class of antioxidant enzymes that are widely found in animals, plants, and microorganisms. They can act as electron acceptors to catalyze the redox reaction between hydrogen peroxide and various electron donors, playing a significant role in the production and clearance of ROS [4,5]. According to their structural characteristics, PRXs can be divided into heme peroxidase and non-heme peroxidase [6]. Among them, non-animal heme peroxidases can be divided into three categories: Class I peroxidases are intracellular enzymes, including microbial cytochrome C peroxidase and ascorbic acid peroxidase [7]; Class II peroxidases are oxidoreductases produced by fungi and belong to extracellular enzymes; and Class III peroxidases (PRXs) are secretory enzymes produced specifically by plants. As a multi-gene family widely distributed among various plants, they are also known as secretory peroxidases [8] due to their ability to secrete into the extracellular space or vacuoles. In plants, PRXs mainly participate in processes such as the clearance of toxic peroxides within the plant body, the synthesis of cell walls, tissue healing, and the synthesis and metabolism of auxins [5,6].

The Prx protein contains highly conserved cysteine residues (-Cys), which are closely related to the antioxidant activity of the enzyme. According to the known Prx gene sequence and amino acid sequence, it can be divided into 1-Cys and 2-Cys, in which *SiPrx* belongs to 2-Cys Prx [9]. 2-Cys Prx can scavenge ROS in cells by oxidizing its conserved cysteine residues [10], and it has been found in many organisms [11,12]. The enzyme contains two highly conserved catalytic cysteine residues, namely Cys52 and Cys172 [13], which are responsible for converting H_2_O_2_ into H_2_O and alcohols in the chloroplasts of higher plants [14,15]. Studies on the structure and function of 2-Cys Prx in Arabidopsis thaliana and Nicotiana benthamiana [16,17] indicate that it can enhance the stress tolerance of plants [18]. Furthermore, genus Brassica plants can also enhance their tolerance to abiotic stress by upregulating the expression of 2-Cys Prx genes [19].

*Silphium perfoliatum* L., native to America, is a perennial herbaceous plant of the Asteraceae family. It is characterized by its high protein content, rapid growth, resistance to pests and diseases, as well as its tolerance to drought and flooding [20]. Consequently, it has the potential to serve as a high-protein quality forage and has begun to be promoted in certain areas of Xinjiang. SP contains a variety of compounds, such as L-ascorbic acid, chlorophylls a and b, tannins, and microelements, including potassium (K), calcium (Ca), magnesium (Mg), zinc (Zn), iron (Fe), etc. These components have extensive application value in the pharmaceutical and food industries [21,22]. Currently, about 16 compounds have been isolated from SP, each with different pharmacological effects [23,24]. Studies have shown that SP extracts have protective effects on the liver, and the extracts from its roots and stems are rich in glycosides with anti-inflammatory and anti-itch properties [25,26]. However, the Xinjiang region has a variety of saline–alkali soils and a wide distribution, which limits the promotion of SP in the Xinjiang area. Therefore, enhancing SP’s resistance to saline–alkali conditions through genetic improvement methods will be of significant importance for the promotion and cultivation of SP in the Xinjiang region and for the development of animal husbandry in Xinjiang.

*Saussurea involucrata*, commonly known as Edelweiss, is mainly distributed in the high-altitude and cold regions of Xinjiang, demonstrating strong cold resistance, radiation resistance, wind and sand resistance, and adaptability to hypoxic environments, making it a unique large-scale higher plant in areas above the Tianshan snow line. Due to the long-term growth of *Saussurea involucrata* in extremely harsh environments, it has evolved numerous unique stress-resistant functional genes, serving as an excellent stress-resistant gene pool and providing high-quality genetic resources for plant molecular breeding; these include *SiFBA5* [27], *SikCOR413PM1* [28], *SiDHN* [29], *Sipip2;7* [30], *Sipip2;4* [31], and *SikPsaF* [32], among others. Previous research has shown that the overexpression of *SiPrx* in tobacco significantly enhances its resistance to abiotic stress [9]. Based on these findings, the current study overexpressed *SiPrx* in SP to investigate its potential to improve the stress resistance of the transgenic plant. The results indicate that *SiPrx* has potential applications in enhancing the cold resistance and salt tolerance of SP. This study offers a research foundation and scientific basis for the exploration of stress-resistant genes in *Saussurea involucrata* and the cultivation of new varieties of SP.

## 2. Materials and Methods

### 2.1. Gene Cloning

Primers specific to the *SiPrx* gene sequence were designed, and corresponding restriction enzyme sites were added to both ends of the primers. The total RNA was extracted from *Saussurea involucrata* using the RNAiso Plus kit (TaKaRa, Biomedical Technology (Beijing) Co., Ltd., Beijing, China) following the manufacturer’s instructions. First-strand cDNA was synthesized from the total RNA using oligo (dT) primers and the Prime Script1 RT enzyme (TaKaRa). PCR amplification was performed using the cDNA as a template, and the purified PCR products were then inserted into the pMD19-T cloning vector and transformed into competent Escherichia coli DH5α cells. Positive clones were screened to extract plasmids for sequencing. Subsequently, the correctly identified *SiPrx* fragment was connected to the plant expression vector pCAMBIA2300 through enzymatic ligation, resulting in the recombinant vector *pCAMBIA2300-35S-SiPrx*, which was then transferred into Agrobacterium tumefaciens GV3101 via electroporation (Supplementary Materials).

### 2.2. Sequence Alignment and Phylogenetic Tree Construction

From the low-temperature transcriptome and genome database maintained by our laboratory, the complete *SiPrx* gene sequence was extracted using the BLAST (The version number is 2.13.0) tool. The extracted gene sequence was translated into a protein sequence, and the corresponding Prx protein sequence information was downloaded from NCBI (https://www.ncbi.nlm.nih.gov/ (accessed on 21 January 2025)). Subsequently, MEGA11 (The version number is 11.0.4) software was used to analyze and align the extracted sequences, and a phylogenetic tree was constructed to display the results.

### 2.3. Genetic Transformation of Silphium perfoliatum L.

The seeds of SP used in this study were provided by the Tori Forage Grass Technology Academy. Selected SP seeds with plump grains were dehulled and then disinfected in a sterile flask using 75% alcohol and 10% sodium hypochlorite in sequence. After disinfection, the seeds were rinsed with sterile water to remove any residual disinfectant. Subsequently, the treated seeds were placed on 1/2 MS medium for cultivation. The sterile explants of SP were transformed using leaf disc transformation with Agrobacterium and then placed on MS medium supplemented with 1.2 mg/L 6-BA + 0.3 mg/L NAA to induce adventitious buds. Once the adventitious buds reached two to three centimeters, they were transferred to 1/2 MS rooting medium supplemented with 0.2 mg/L NAA for rooting induction. After the regenerated plants developed robust root systems, they were transplanted into pots for further cultivation. Once the regenerated plants produced three new leaves in the pot, their genomic DNA was extracted, and the transgenic plants were identified using DNA-PCR and RT-PCR (Supplementary Materials).

### 2.4. Stress Treatment of Plants

Six WT and six transgenic plants with similar sizes were selected and cultivated in a tissue culture room under conditions of 24 °C, a 12 h light/dark cycle, 60–70% relative humidity, and a light intensity of 30,000 lx. The transgenic and WT plants were divided into two groups, each with three replicates, and the roots were irrigated with 500 mL of 200 and 300 mmol/L NaCl solution for one week, respectively (through gradient experiments, we found that WT SP exhibits wilting and death phenomena when treated with the 250 mmol/L NaCl solution; experimental results figure refers to the Supplementary Materials). The morphological changes of the plants before and after treatment with the resin canary grass were recorded; fresh leaf samples were collected before and after treatment, frozen in liquid nitrogen, and stored in a −80 °C ultra-low-temperature freezer for future use.

Three WT and three transgenic plants of similar size were selected, placed in an outdoor low-temperature environment (average temperature −10 °C) for 48 h, and then returned to the indoor environment to record their status. The morphological changes of the plants before and after treatment with the resin of the Comarum palustre were recorded and photographed, fresh leaf samples were collected before and after treatment, and the samples were frozen in liquid nitrogen for storage in a −80 °C freezer for future use.

### 2.5. SiPrx Gene Expression Analysis Under Stress Conditions

First, 2.4 processed leaf samples were removed from the −80 °C ultra-low-temperature freezer. The same RNA extraction and reverse transcription methods were used as described in Section 2.1 to prepare cDNA from the processed leaf samples, serving as templates for qRT-PCR, and they were standardized using *GAPDH* as the reference gene. We calculated the relative expression levels of each gene using the 2^−ΔΔCt^ method.

### 2.6. Physiological and Biochemical Index Determination

The relative water content (RWC) was measured using the weighing method. The content of malondialdehyde (MDA) in the sample was determined using the thiobarbituric acid method [33]. The content of Free Proline (Pro) in the sample was determined via ninhydrin colorimetry [34]. The relative conductivity (REC) was measured with an EC215 conductivity meter.

Extraction of crude enzyme solution: After precisely weighing 0.5 g of the sample, homogenization was performed under low-temperature conditions using a solution containing 1.0 mmol EDTA-Na2 and a 2% (*w*/*v*) polyvinylpyrrolidone in 50 mmol per liter cold phosphate buffer (pH 7.8). After grinding, the crude enzyme solution was extracted via low-temperature centrifugation. Finally, the enzyme activities of superoxide dismutase (SOD), peroxidase (POD), and catalase (CAT) were determined using spectrophotometry [35] (considering the complexity of the measurement method, this article does not elaborate it in detail in the main text; for specific steps, please refer to the Supplementary Materials). The glutathione peroxidase (GPX) enzyme activity was determined using a GPX activity assay kit (Beijing, China, Solarbio, GSH-Px/GPX Activity Assay Kit, Model: BC1195), and the operation was carried out according to the instructions provided.

### 2.7. Data Processing and Analysis

All the data were analyzed and graphically represented using GraphPad Prism 8 (GraphPad Software, San Diego, CA, USA). The statistical analyses were conducted using SPSS 26 (IBM, Inc., Armonk, NY, USA). Student’s *t*-test was used to compare the statistically significant differences (*p* ≤ 0.05) between the means of the parameters tested. All the data are presented as the means of three biological replicates.

## 3. Results and Analysis

### 3.1. Sequencing and Analysis of the Phylogenetic Tree of SiPrx

**Figure 1:**
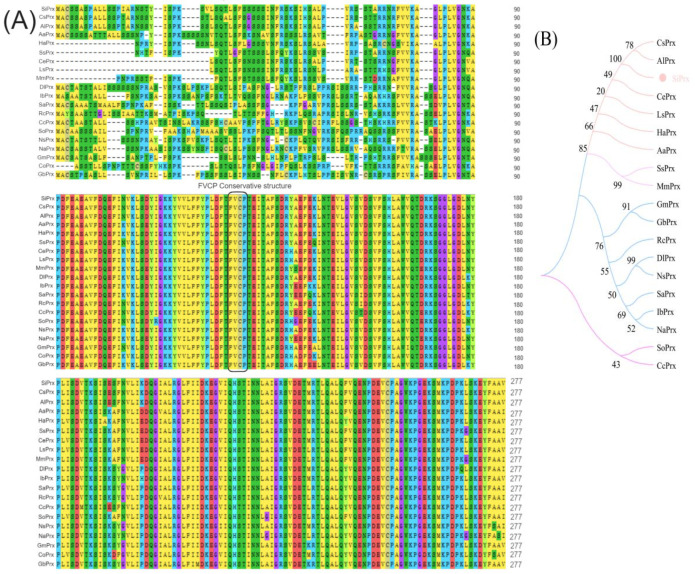
SiPrx BAS1 sequence and phylogenetic tree analysis. (**A**) SiPrx BAS1 sequence structure analysis; (**B**) SiPrx phylogenetic tree analysis. (CsPrx: XP_024962007.1:1-263 2-Cys peroxiredoxin BAS1, chloroplastic-like; AlPrx: KAI3696851.1:1-263 hypothetical protein L6452_29435; CePrx: KAI3511992.1:30-265 hypothetical protein L1887_19154; AaPrx: PWA51182.1:1-267 thioredoxin-like fold protein; HaPrx:XP_021984163.1:14-260 2-Cys peroxiredoxin BAS1, chloroplastic; LsPrx: XP_023768080.1:30-265 2-Cys peroxiredoxin BAS1, chloroplastic; SsPrx: KAI3716609.1:15-257 hypothetical protein L1987_67599; MmPrx: KAD5508523.1:15-261 hypothetical protein E3N88_16226; SoPrx: XP_021867340.1:1-270 2-Cys peroxiredoxin BAS1, chloroplastic; IbPrx: GMD39626.1:1-270 2-Cys peroxiredoxin BAS1, chloroplastic; NaPrx: XP_019239930.1:1-271 PREDICTED: 2-Cys peroxiredoxin BAS1, chloroplastic; SaPrx: KAK4432617.1:1-265 2-Cys peroxiredoxin BAS1-like, chloroplastic; CcPrx: KAF9597756.1:2-263 hypothetical protein IFM89_021510; RcPrx: XP_002530152.1:1-266 2-Cys peroxiredoxin BAS1, chloroplastic; DlPrx: XP_052176104.1:1-271 2-Cys peroxiredoxin BAS1, chloroplastic-like; NsPrx: KAA8518506.1:1-267 hypothetical protein F0562_015980; GmPrx: NP_001341836.1:1-258 2-cys peroxiredoxin; GbPrx: XP_061353561.1:1-265 2-Cys peroxiredoxin BAS1, chloroplastic).

Through the alignment analysis of multiple 2-Cys Prx protein sequences, it was discovered that the SiPrx protein sequence contains a conserved sequence composed of phenylalanine–valine–cysteine–proline (FVCP), and SiPrx also possesses two lysine residues (Figure 1A), indicating that SiPrx belongs to the 2-Cys Prx protein family. Evolutionary analysis results show that although SiPrx is closely related to the Prx proteins in *Cynara scolymus* L. and *Arctium lappa* L. and has a high degree of homology (Figure 1A), it is located on different branches in the evolutionary tree (Figure 1B), suggesting that the *SiPrx* gene from *Saussurea involucrata* may have its unique functions.

### 3.2. Analysis of SiPrx Expression Under Stress Conditions

**Figure 2:**
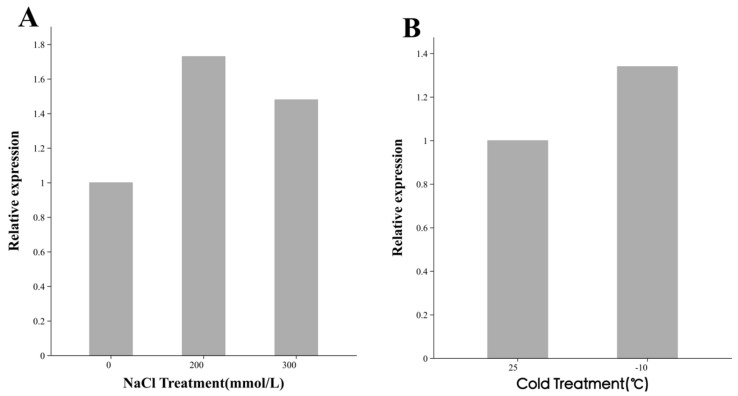
Analysis of relative expression levels of *SiPrx* before and after stress treatment. (**A**) Analysis of relative expression levels of *SiPrx* before and after salt treatment. (**B**) Analysis of relative expression levels of *SiPrx* before and after low-temperature treatment.

To evaluate whether the expression level of *SiPrx* in transgenic SP is affected by stress conditions, we determined the differences in *SiPrx* gene expression levels in the leaves of transgenic SP under various concentrations of salt stress and low-temperature stress conditions. Since the recombinant vector *pCAMBIA2300-35S-SiPrx* uses the strong 35S promoter, gene expression levels are generally high, and therefore, the relative expression level differences are not particularly pronounced. The qRT-PCR results show that after salt stress treatment, the expression level of the SiPrx gene in transgenic plants exhibited an upregulation trend compared to before treatment, reaching a maximum expression level at a concentration of 200 mmol/L, while at 300 mmol/L, there was a slight downward trend in its expression. This may be due to the excessively high salt concentration causing damage to intracellular components due to severe dehydration, thereby reducing gene expression (Figure 2A). Similarly, under cold stress treatment, the relative expression level of the *SiPrx* gene in transgenic plants was also higher than that under room temperature conditions (Figure 2B). These results indicate that the *SiPrx* gene in transgenic SP can respond to salt and cold stress and participates in the plant stress defense mechanism by upregulating its expression level.

### 3.3. Overexpression of SiPrx Enhances the Salt Tolerance of Silphium perfoliatum L.

**Figure 3:**
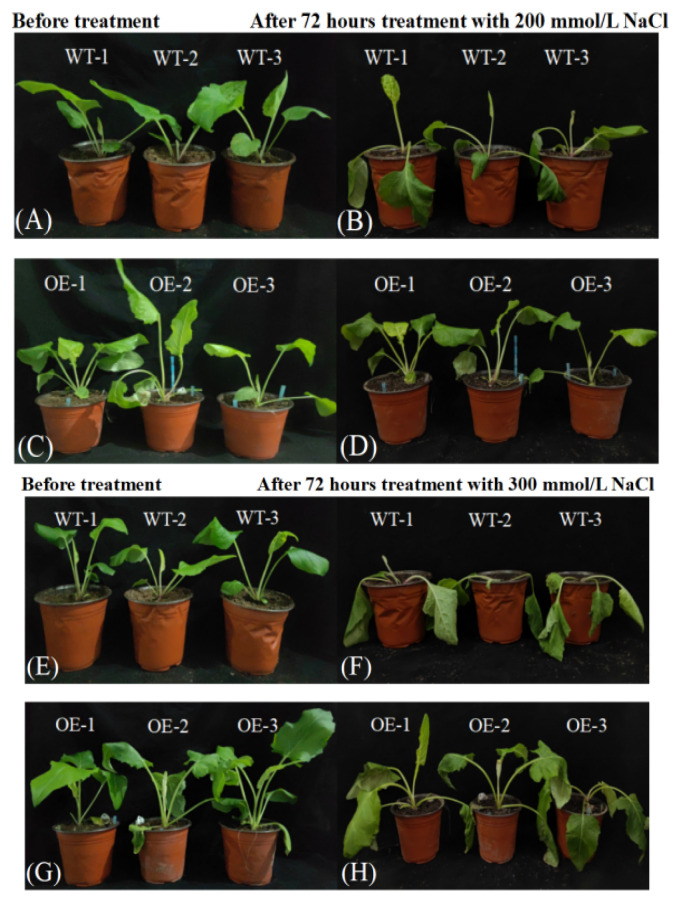
Phenotypic comparison of *Silphium perfoliatum* L. after 72 h of NaCl stress treatment. (**A**) Wild SP plants before treatment with 200 mmol/L NaCl; (**B**) WT after treatment with 200 mmol/L NaCl for 72 h; (**C**) transgenic SP plants before treatment with 200 mmol/L NaCl; (**D**) transgenic SP plants after treatment with 200 mmol/L NaCl for 72 h. (**E**) Wild SP plants before treatment with 300 mmol/L NaCl; (**F**) WT after treatment with 300 mmol/L NaCl for 72 h; (**G**) transgenic SP plants before treatment with 300 mmol/L NaCl; (**H**) transgenic SP plants after treatment with 300 mmol/L NaCl for 72 h.

Before salt treatment, there were no significant differences in the appearance and growth status between WT and transgenic SP (Figure 3A,C,E,G). After treatment with 200 mmol/L NaCl solution for 72 h, although the peripheral leaves of WT SP began to bend from the base, showing morphological characteristics of lodging, the leaves still maintained a relatively full state, while the leaves located in the center of the plant did not show significant morphological changes (Figure 3B). Under the same treatment conditions, the leaves of transgenic SP did not show lodging, the small peripheral leaves showed withering, but the rest of the normal-sized leaves did not show significant differences compared to before treatment (Figure 3D). After treatment with 300 mmol/L NaCl solution for 72 h, the leaves of WT SP showed complete withering, indicating that 300 mmol/L NaCl had reached its lethal concentration (Figure 3F); under the same treatment conditions, only some leaves of transgenic SP showed slight withering, and its growth status remained good, which fully indicated that 300 mmol/L NaCl had not reached its lethal concentration (Figure 3H).

**Figure 4:**
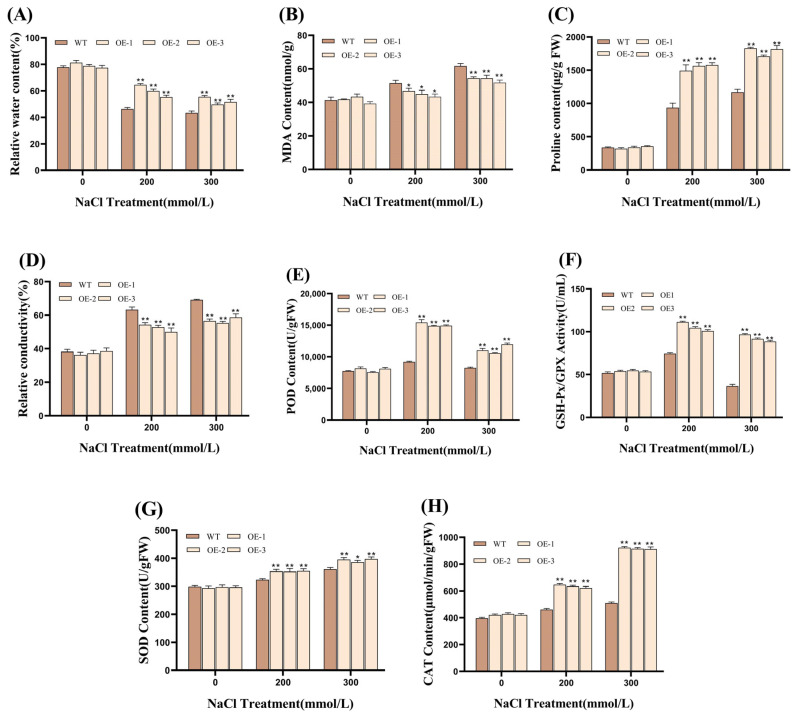
Analysis of physiological indicators and antioxidant enzyme activities in *Silphium perfoliatum* L. treated with NaCl for 72 h. (**A**) Relative water content; (**B**) MDA; (**C**) proline content; (**D**) relative conductivity; (**E**) POD; (**F**) GPX; (**G**) SOD; (**H**) CAT. (* *p* < 0.05 and ** *p* < 0.01 for comparisons between the transgenic lines and WT plants using Student’s *t*-tests).

Furthermore, physiological and biochemical indicators showed that the RWC of WT and transgenic plants showed a downward trend after salt stress, but the decline in transgenic plants was significantly lower than that in WT, and the RWC of transgenic plants was always higher than that of WT. Under the condition of 200 mmol/L NaCl, the RWC of transgenic plants was about 29.09% higher than that of WT. When the NaCl concentration increased to 300 mmol/L, the difference decreased to about 20.2% (Figure 4A), which indicated that transgenic plants had a stronger ability to maintain water homeostasis and metabolic processes under environmental stress. As a marker of lipid peroxidation in cell membranes, the MDA content directly reflects the degree of cell membrane damage. Under NaCl treatment, the MDA content of the WT and transgenic plants increased, but the MDA content of the transgenic plants was always lower than that of the WT plants. Under the treatment of 200 mmol/L NaCl, the MDA content of the transgenic plants decreased by about 14.25% compared with that of the WT plants. Under the treatment of 300 mmol/L NaCl, the MDA content of the transgenic plants decreased by about 16.78%, which indicated that the cell membrane damage of transgenic plants was always lower. (Figure 4B). The results of Pro content determination showed that the Pro accumulation of transgenic plants under NaCl treatment was significantly higher than that of WT. Under the treatment of 200 mmol/L NaCl, the Free Proline content of the transgenic plants was about 64.21% higher than that of the WT. Under the treatment of 300 mmol/L NaCl, it was about 52.91% higher (Figure 4C), which indicated that the transgenic SP had stronger osmotic adjustment ability. The results of REC determination showed that the REC of transgenic plants was always lower than that of WT plants under NaCl treatment, and the REC of transgenic plants was reduced by about 20.66% compared with WT plants under 200 mmol/L NaCl treatment. Under the treatment of 300 mmol/L NaCl, the REC decreased by about 21.42%, indicating that it has higher cell membrane stability (Figure 4D). The results of enzyme activity determination showed that the activities of SOD, GPX, POD, and CAT in transgenic plants were significantly higher than those in WT plants under NaCl treatment, which indicated that transgenic SP had stronger antioxidant capacity and could resist oxidative damage caused by salt stress more effectively (Figure 4E–H).

### 3.4. Overexpression of SiPrx Enhances the Freezing Tolerance of Silphium perfoliatum L.

**Figure 5:**
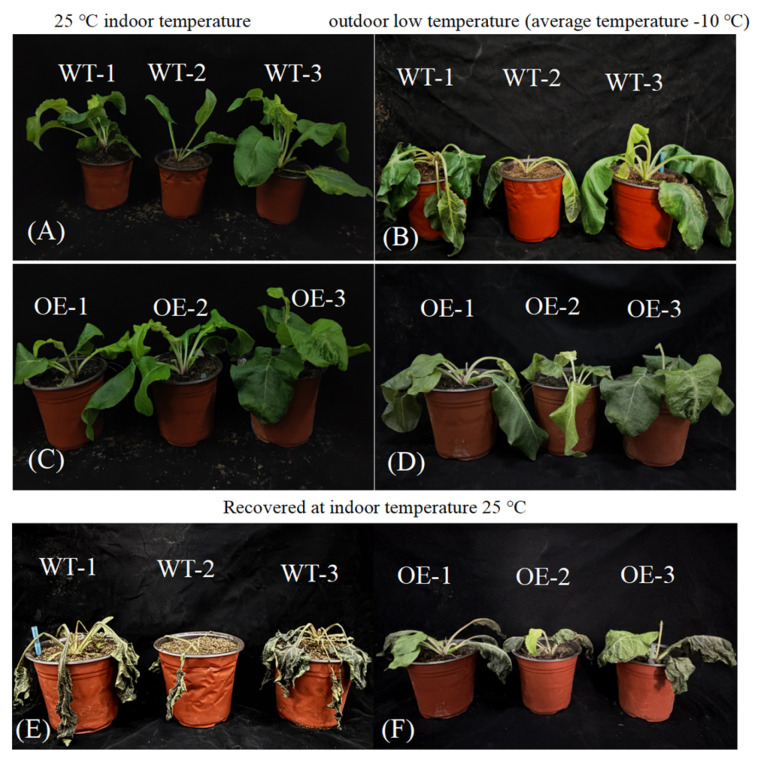
Phenotypic comparison of *Silphium perfoliatum* L. before and after 48 h of outdoor cold treatment and after room temperature recovery. (**A**) Wild SP plants growing normally at an indoor temperature of 25 °C; (**B**) wild SP plants after 48 h of low-temperature treatment outdoors; (**C**) transgenic SP plants growing normally at an indoor temperature of 25 °C; (**D**) transgenic SP plants after 48 h of low-temperature treatment outdoors; (**E**) wild SP plants recovering indoors for 72 h after outdoor low-temperature treatment; (**F**) transgenic SP plants recovering indoors for 72 h after outdoor low-temperature treatment.

At a constant temperature of 25 °C, there was no obvious difference in the appearance between the WT and the transgenic plants, and their leaves were full and bright green (Figure 5A,C), indicating that the overexpression of the *SiPrx* gene did not significantly affect the growth of the transgenic plants.

However, after 48 h of outdoor low-temperature treatment, the leaves of WT SP showed an obvious wilting phenomenon, accompanied by obvious dark-black freezing injury symptoms. However, under the same conditions, only some leaves of the transgenic SP wilted slightly, and the whole plant still maintained a good growth state (Figure 5B,D). At a room temperature of 25 °C, after 72 h of storage, the leaves of the WT SP were completely dry and necrotic, and the failed resuscitation attempt led to death. Nearly half of the leaves in the transgenic SP showed necrosis and dryness; however, the veins of these necrotic leaves remained full. The other leaves maintained a good growth state and showed a healthy growth trend as a whole (Figure 5E,F).

**Figure 6:**
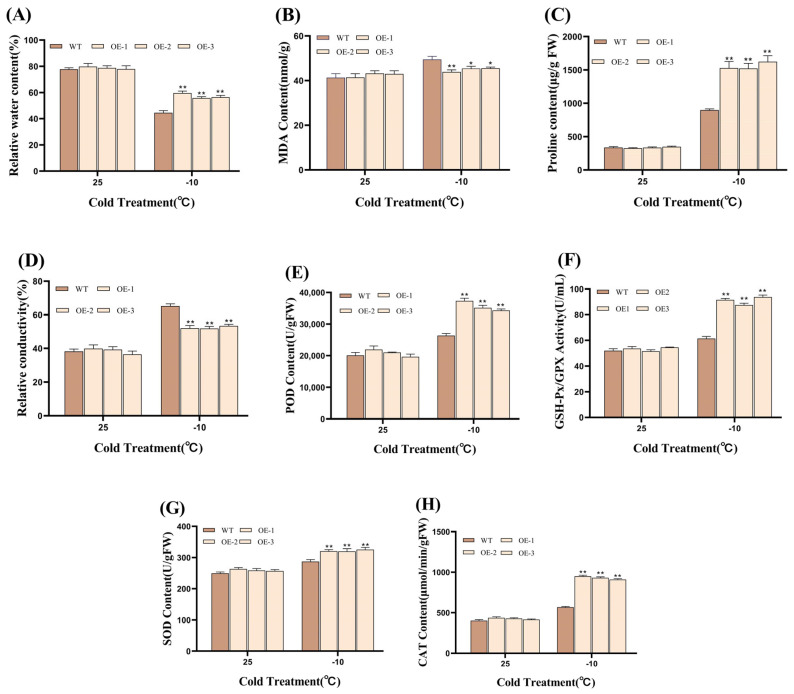
Analysis of physiological indicators and antioxidant enzyme activities in *Silphium perfoliatum* L. after 48 h of low-temperature treatment. (**A**) Relative water content; (**B**) MDA; (**C**) proline content; (**D**) relative conductivity; (**E**) POD; (**F**) GPX; (**G**) SOD; (**H**) CAT. (* *p* < 0.05 and ** *p* < 0.01 for comparisons between the transgenic lines and WT plants using Student’s *t*-tests).

The results of the physiological and biochemical indexes showed that the RWC of transgenic plants after low-temperature treatment was about 24.38% higher than that of WT, and the higher RWC indicated that transgenic plants could maintain cellular water more effectively under low-temperature stress (Figure 6A). The determination of MDA content showed that the MDA content of the transgenic plants decreased by about 10.09% compared with that of the WT at a low temperature, which indicated that the degree of lipid peroxidation of the cell membrane of transgenic Pinus sylvestris was low and that the degree of cell membrane damage was light (Figure 6B). At the same time, the results of Pro determination showed that the accumulation in transgenic plants was significantly higher than that in WT, reaching 73.59%, which further revealed that the transgenic plants had stronger osmotic adjustment ability than the WT plants (Figure 6C). The determination results of the REC showed that the REC of transgenic plants decreased by about 24.33% compared with the WT plants under low-temperature treatment, indicating that their cell membranes were more stable (Figure 6D). The results of related antioxidant enzyme activity determination showed that the activities of SOD, POD, CAT, and GPX in transgenic plants were significantly higher than those in WT plants under low-temperature treatment, which indicated that transgenic Vanilla alternifolia had stronger antioxidant capacity and could resist oxidative damage caused by low-temperature stress more effectively (Figure 6E–H).

Under low-temperature stress, although transgenic SP experienced necrosis and desiccation in some leaves after low-temperature treatment, the veins of these necrotic leaves remained plump, indicating that the expression of *SiPrx* protected the integrity of the vein tissue to some extent. As the main channel for the transport of water and nutrients within the plant, the maintenance of vein integrity is crucial for the survival and recovery of plants under adverse conditions. The above results suggest that the overexpression of the *SiPrx* gene in SP can significantly enhance its freezing tolerance.

## 4. Discussion

In 1996, scientists first discovered the plant *Prx* gene in barley and named it *Hv-1-CysPrx* and *Hv-2-CysPrx* [36]. Since then, this gene has been found in many species, including spinach, gentian, and Arabidopsis thaliana [37,38]. According to the number and position of Cys residues, Prxs can be divided into five subtypes: 1-Cys Prxs, 2-Cys Prxs, PrxII, PrxQ, and Grx (glutathione peroxidases) [39]. Among them, 2-Cys Prxs is the most conservative, and the sequence analysis of this study also confirms this view. SiPrx shows high homology with the 2-Cys Prxs of globe artichoke and great burdock.

Research has found that Prx proteins play a significant role in protecting the body from oxidative damage and enhancing immune responses [40]. Prx possesses the functions of eliminating ROS within organisms, regulating intracellular signal transduction, and acting as a molecular chaperone [41,42,43]. The most widely studied aspect is its function in eliminating ROS. Prx has a high affinity for H_2_O_2_, ensuring its effective removal. The mechanism involves the oxidation of the N-terminal cysteine residue to Cys-SOH by H_2_O_2_, which then forms an intramolecular disulfide bond by combining with the Cys-SH of an adjacent subunit. This disulfide bond can be reduced by Trx. Simultaneously, H_2_O_2_ is reduced to H_2_O by Prx and ultimately converted to ethanol, which is then cleared out of the cell. Yuan et al. [44] revealed that PrxII and 2-Cys Prxs cloned from *Dunaliella viridis* were involved in the process of salt stress through yeast experiments. Contreras et al. [45] found that under copper stress, the Prx protein in *Scytosiphon gracilis* is upregulated. Jing Liwen [46] found that the overexpression of AtPrx under NaCl, KCl, high-temperature, low-temperature, H_2_O_2_, and other stress conditions was helpful to improve the stress resistance of yeast. Nostoc flagelliforme can sense external drought stress and reduce the cell damage caused by drought by overexpressing the NfPrx protein [40]. *Candidatus* liberibacter asiaticatus (CLas) can eliminate excess H_2_O_2_ by increasing the expression of CLasPrx protein, thus avoiding oxidative damage [47]. In this study, the *SiPrx* gene was cloned from *Saussurea involucrata* and expressed in different sources. After stress treatment, through the detection of *SiPrx* expression in transgenic plants, the observation of the plant phenotype, and the determination of related physiological and biochemical indexes, the transgenic plants after stress treatment showed an upward trend of *SiPrx* gene expression, and at the same time, the excessive expression of *SiPrx* gene also significantly improved the tolerance of SP to salt and low temperatures.

We utilized phenotypic observations and internal physiological and biochemical changes in wild and transgenic *Silphium perfoliatum* L. to comprehensively evaluate the resistance of overexpressing *SiPrx* SP. The RWC is an index to measure the metabolic intensity of plants [48], and Pro is a component of plant proteins, which usually exists in plants in a free state [49]. The Free Proline content in plants reflects their tolerance to adversity. Transgenic SP showed higher RWC and Pro levels than WT SP under high-salt and low-temperature conditions, which indicated that the overexpression of *SiPrx* enhanced the osmotic adjustment ability and metabolic activity of transgenic SP under high-salt and high-osmotic-pressure and low-temperature conditions. The REC is an index to evaluate the degree of cell membrane damage in plants [50], while the MDA content is a key index to measure the degree of cell damage, which reflects the potential antioxidant capacity of organisms [51]. MDA not only represents the rate and severity of lipid peroxidation but also indirectly indicates the degree of tissue damage [52]. Generally, combining the MDA level with the REC can effectively reveal the damage status of the plant cell membrane [53]. In a high-salt and low-temperature environment, the MDA content and REC of transgenic SP are obviously lower than that of the WT plant, which indicates that transgenic SP has stronger ROS scavenging ability than wild plants and can minimize the degree of membrane lipid peroxidation caused by ROS. The stress resistance of plants can be evaluated by measuring the activity levels of CAT, SOD, POD, and GPX [54,55,56,57,58,59]. To further confirm the enhancement effect of *SiPrx* overexpression on the antioxidant capacity of transgenic SP, we compared the changes in the CAT, SOD, POD, and GPX activities in transgenic SP and WT plants under high-salt and low-temperature conditions. The results showed that under 200 mmol/L NaCl conditions, the CAT, SOD, POD, and GPX activities of transgenic SP reached 1.37 times, 1.09 times, 1.63 times, and 1.35 times that of the WT, respectively; under 300 mmol/L NaCl conditions, these activities increased to 1.41 times, 1.08 times, 1.35 times, and 2.51 times, respectively; and under −10 °C conditions, the activities increased to 1.63 times, 1.12 times, 1.34 times, and 1.48 times, respectively. Especially following the outdoor freezing treatment, all three transgenic SP plants survived, while all WT SP plants perished. This more clearly illustrates the robust survival ability of transgenic SP in harsh environments, confirming the enhancement of SiPrx on the stress resistance of transgenic SP plants. These physiological and biochemical indicators consistently indicate that the overexpression of *SiPrx* significantly enhances the antioxidant capacity of transgenic SP and its ability to survive in harsh environments.

In summary, transgenic SP overexpressing *SiPrx* exhibits stronger salt tolerance and low-temperature tolerance than WT plants. We believe that when the cell membranes of transgenic SP plants are stimulated by external factors and undergo peroxidation, SiPrx will play its role in regulating intracellular signal transduction and molecular chaperone functions to clear excessive ROS in the body, thereby enhancing the stress resistance of the transgenic plants.

## 5. Conclusions

In this study, we successfully introduced the *SiPrx* gene of *Saussurea involucrata* into the herb, and through a series of physiological and biochemical analyses, we verified the remarkable effect of the *SiPrx* gene in improving the salt tolerance and freezing resistance of the herb. Under the condition of salt stress, transgenic SP showed higher antioxidant enzyme activity, lower MDA content, and higher proline content, which could better maintain the stability and integrity of its cell membranes. Similarly, under low-temperature stress, the transgenic plants also showed stronger antioxidant capacity and a lower degree of cell membrane damage. These findings not only confirm the important role of *SiPrx* gene in plants to cope with abiotic stress but also provide new ideas and methods for improving crop stress resistance via genetic engineering technology. In addition, this study also revealed the new function of *SiPrx* in regulating the plant antioxidant system, especially the significant increase in GPX activity, which provided a new perspective for further understanding the stress response mechanism of plants. To sum up, *SiPrx* gene has great application potential in the salt tolerance and frost resistance of plants, and its molecular mechanism will be further explored in the future in order to play a greater role in agricultural and forestry production.

## Data Availability

No new data were created or analyzed in this study.

## Supplementary Materials

The following supporting information can be downloaded at https://www.mdpi.com/article/10.3390/plants14071030/s1: Figure S1: Experiment on gradient growth of wild type *Silphium perfoliatum* L. with NaCl irrigation; Figure S2: *SiPrx* Agrobacterium colony PCR; Figure S3: DNA-PCR; Supplementary S1: Enzyme activity assay method; Supplementary S2: *SiPrx* ORF.
