# Supplementary material for: The Gene SiPrx from Saussurea involucrata Enhances the Stress Resistance of Silphium perfoliatum L
Source: Plants (Basel). 2025 Mar 26;14(7):1030. doi: 10.3390/plants14071030 (PMC11990491; doi:10.3390/plants14071030)
Supplement: Supplementary file 1 [file plants-14-01030-s001.zip › plants-3520394-supplementary.pdf]

Supplementary materials

THE Gene *SiPrx* from *Saussurea involucrata* Enhances the Stress Resistance of *Silphium perfoliatum* L.

Tao Liu[a,1], Baotangh Wu[a,1], Yao Zhang[a], Zhongqing Li[a], Yanhua Xue[a], Xiaoqin Ding[a] , Zhihui Yang[a] , Jianbo Zhu[a*], Yajie Han[b*]

[a] College of Life Science, Shihezi University, Shihezi 832003, People's Republic of China.

[b]School of Chemistry and Chemical Engineering, Shihezi University, Shihezi 832003, People's Republic of China.

Corresponding Author:

Jianbo Zhu

Tel: 86993- 2057515, Fax: 86993-2057262, E-mail address: zhujianboshz@163.com.

Yajie Han

Tel: 86993- 2057515, Fax: 86993-2057270, E-mail address: yaji1978@126.com.

[1] These authors contributed equally to this work.

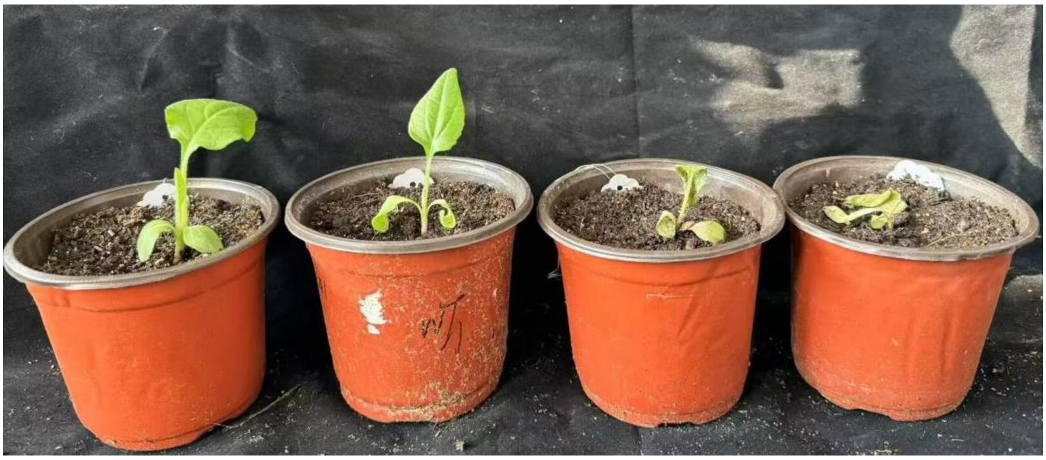

Figure S1: Experiment on gradient growth of wild type *Silphium perfoliatum* L. with NaCl irrigation(The two pots on the left are irrigated with a concentration of 200mmol/L; the two pots on the right are irrigated with a concentration of 250mmol/L.)

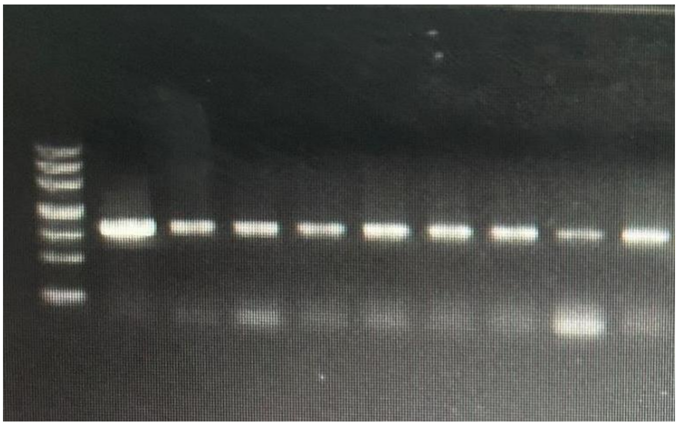

Figure S2: *SiPrx* Agrobacterium colony PCR(The sizes of the indicator strip bands from left to right are 200bp, 500bp, 800bp, 1200bp, 2000bp, and 3000bp respectively.)

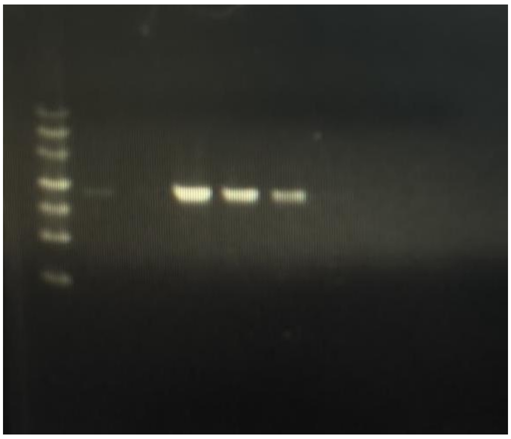

Figure S3: DNA-PCR(The sizes of the indicator strip bands from left to right are 200bp, 500bp, 800bp, 1200bp, 2000bp, and 3000bp respectively.)

(1) SOD: Take 5 transparent test tubes, 3 for sample determination, 1 for the control, and another for the blank. Add 1.5ml of 0.05mol/L PBS buffer solution, 0.3ml of 130mmol/L methionine (Met) solution, 0.3ml of 750umol/L nitroblue tetrazolium (NBT) solution, 0.3ml of 100umol/L EDTA-Na2 solution, 0.3ml of 20uM riboflavin solution, and 0.05ml of enzyme solution (use buffer solution instead of the blank and control tubes) to each tube, and 0.25ml of distilled water. After mixing the above solutions, place the blank tube in a dark place, and the other tubes under a 4000x fluorescent lamp for a 20-minute reaction (ensure that all tubes are exposed to light uniformly; the reaction time can be appropriately shortened when the temperature in the reaction chamber is high and can be extended when the temperature is low). Remember to add riboflavin quickly and avoid direct sunlight exposure (in darkness).

After the SOD activity determination and calculation, use the tube not exposed to light as the blank, and measure the extinction values at 560nm wavelength for the other tubes. The SOD activity unit is defined as the amount of enzyme activity that inhibits 50% of the photochemical reduction of NBT. Calculate the SOD activity using the following formula:

SOD total activity = (A0 - As) × Vt / (A0 × 0.5 × FW × V1)

Where the SOD total activity is expressed as enzyme units per gram of fresh weight; specific activity is expressed as enzyme units/mg protein;

A0 - Extinction value of the light-exposed control tube;

As - Extinction value of the sample tube;

Vt - Total volume of the sample solution (ml);

V1 - Sample volume used during measurement (ml);

FW - Sample weight (g);

Protein concentration is expressed as milligrams of protein per gram of fresh weight (mg/g).

(2) POD: Transfer the supernatant into a 100ml volumetric flask, dilute to the mark with phosphate buffer, and store in a cool place for later use.

Take three test tubes, one with 3ml of reaction solution and add 1ml of phosphate

buffer for zero adjustment, two with 3ml of reaction solution and add 1ml of enzyme solution, start timing immediately, measure at 470nm, and read the enzyme every 30 seconds.

Enzyme activity calculation: An increase of 0.01 in OD value per minute is defined as one unit of enzyme activity (u).

POD = (ΔA470 * Vt) / (W * Vs * 0.01 * t) (ug min)

ΔA470: Change in absorbance during the reaction time; W is the fresh weight of the sample (g); t is the reaction time (min); Vt is the total volume of enzyme solution extracted, 2ml; Vs is the volume of enzyme solution used for measurement.

(3)CAT: Obtain three 10ml test tubes, with two serving as sample measurement tubes and one as a blank tube (boil in boiling water for 5-10 minutes after adding enzyme solution, then cool and add H2O2 to measure the absorbance), add crude enzyme solution 0.2ml, PBS (pH7.8) 1.5ml, and distilled water 1.0ml to each tube in sequence.

After preheating at 25°C, add 0.3ml of 0.1mol/L H2O2 to each tube one by one, start timing immediately after adding to each tube, and quickly pour into a quartz cuvette for absorbance measurement at 240nm. Read the value once every minute for 4 minutes. After all three tubes have been measured, calculate the enzyme activity using the following formula.

Calculation of Results:

An enzyme amount that reduces A240 by 0.1 in 1 minute is defined as 1 enzyme activity unit (u).

Catalase activity (u/g/min) = A240 × Vt / 0.1 × V1 × t × FW

Where A240 = (AS0 - (AS1 + AS2)) / 2

AS0 - Absorbance value of the control tube with inactivated enzyme solution;

AS1, AS2 - Absorbance values of the sample tubes;

Vt - Total volume of crude enzyme extract (ml);

V1 - Volume of crude enzyme solution used for measurement (ml);

FW - Fresh weight of the sample (g);

0.1 - A decrease of 0.1 in A240 corresponds to 1 enzyme activity unit (u);

t - Time from adding hydrogen peroxide to the last reading (min).

Supplementary S1: Enzyme activity assay method

ATGGCTTGTTCATCTGCTTCACCTGCTCTTCTTTCTTCTC

CAATCGCTAGAAACTCCACTTACATTTCCCCCAAATCCG

TTCTCTCCCAAACCCTAAGTTTTTCCAGTTCTTCCTCGA

TCAATTTCAGATCCAAATCCATCCACTCCGCACTCCCCG

TTCGCTCTTCTACCGCTCGTCGCAATCGATTCGTTGTCA

AGGCTGGACTACCACTAGTTGGAAACAAGGCACCAGA

CTTCGAAGCAGAAGCCGTTTTTGATCAAGAGTTCATCA

ATGTTAAGCTCTCTGATTATATCGGGAAGAAATATGTGG

TACTCTTCTTCTACCCATTGGACTTCACTTTTGTTTGTCC

AACTGAGATCACTGCTTTTAGCGACCGATATGCTGAATT

TGAGAAGTTGAACACAGAAGTATTGGGTGTTTCTGTAG

ACAGCGTGTTCTCGCATCTTGCTTGGGTACAAACAGAT

AGAAAGTCTGGGGGCCTTGGTGATTTGAACTATCCATT

GATTTCGGATGTGACAAAGTCAATTTCGGAGTCGTTTAA

TGTGTTGATCAAAGATCAGGGGATAGCGTTGAGAGGGC

TATTCATAATAGACAAGGAAGGGGTTATTCAGCACTCG

ACGATCAACAATCTTGCAATCGGCAGAAGCGTAGATGA

AACAATGAGAACACTTCAGGCATTGCAATTTGTACAAG

AGAACCCAGATGAGGTATGCCCAGCTGGATGGAAGCCT

GGGGAGAAGTCAATGAAGCCAGATCCTAAACTCAGCA

AGGAATACTTTGCAGCTGTATAA

Supplementary S2: *SiPrx* ORF
